# Oxytocin receptor antagonism in migraine: a randomized, double-blind, placebo-controlled provocation study

**DOI:** 10.1186/s10194-026-02297-z

**Published:** 2026-02-16

**Authors:** Mira Pauline Fitzek, Paula Kleist, Cleo Handtmann, Lucas Hendrik Overeem, Carolin Luisa Hoehne, Kristin Sophie Lange, Cornelius Angerhöfer, Yones Salim, Andreas D. Ebert, Nicole Mattern, Uwe Reuter, Bianca Raffaelli

**Affiliations:** 1 Department of Neurology, Charité Universitätsmedizin Berlin, Charitéplatz 1, 10117 Berlin, Germany; 2 (Junior) Clinician Scientist Program, Berlin Institute of Health (BIH), Berlin, Germany; 3 Praxis für Gynäkologie & Geburtshilfe, Nürnberger Str. 67, 10787 Berlin, Germany; 4 Praxis für Gynäkologie & Geburtshilfe, Schloßstraße 22, 13507 Berlin, Germany; 5 Universitätsmedizin Greifswald, Greifswald, Germany

**Keywords:** Trigger, Hypothalamus, Vascular response, Vasodilatation, Migraine

## Abstract

**Background and objectives:**

Oxytocin has been implicated in migraine pathophysiology through its roles in pain modulation and vascular regulation. Its receptors are present in migraine-related brain regions and cerebral vessels and interact with key migraine mediators such as calcitonin gene-related peptide (CGRP). Declining oxytocin levels coincide with increased migraine frequency, but a causal role in humans remains unclear. This study examined whether acute oxytocin receptor blockade provokes migraine attacks or alters cerebrovascular function.

**Methods:**

In this randomized, double-blind, placebo-controlled, cross-over study, women with episodic migraine (WM), healthy women (HC), and men with episodic migraine (MM) aged 18–45 years received the oxytocin receptor antagonist atosiban (6.75 mg bolus followed by 54 mg over 3 h) or placebo on two separate visits. Women used continuous hormonal contraception to ensure stable hormone levels. The primary endpoint was the incidence of migraine-like attacks in WM within 12 h post-infusion. Secondary endpoints included the incidence and intensity of any headache and vascular responses.

**Results:**

A total of 20 WM (27.1 ± 5.6 years), 20 HC (25.7 ± 6.8 years) and 20 MM (29.3 ± 6.1 years) completed both provocations visits. During the 12-hour observation period, the incidence of migraine-like attacks in WM did not differ between atosiban and placebo (atosiban: 6 (30%) vs. placebo: 4 (20%; *p* = 0.75)). Headache of any kind were reported in 15 (75%) vs. 11 (55%) (*p* = 0.34). HC did not experience migraine-like attacks, though nine (45%) participants reported headache of any kind after atosiban vs. six (30%) after placebo (*p* = 0.25). Among MM, migraine attacks occurred in two (10%) participants after atosiban vs. three (15%) after placebo (*p* > 0.999). While HC showed significantly increased temporal artery diameter and middle cerebral artery flow velocity during atosiban administration, no significant vascular changes were observed in participants with migraine.

**Discussion:**

Short-acting oxytocin receptor antagonism did not trigger migraine attacks, suggesting that acute suppression of oxytocin signaling alone is unlikely to trigger migraine under stable hormonal conditions. Instead, oxytocin may act as a modulatory factor within neurovascular networks. Future studies using longer-lasting or brain-penetrant antagonists are needed to further clarify oxytocin’s role in migraine susceptibility.

**Trial registration information:**

DRKS – Deutsches Register Klinischer Studien: DRKS00033341. Registered: 08.01.2024. First patient enrolled: 08.07.2024.

**Supplementary Information:**

The online version contains supplementary material available at 10.1186/s10194-026-02297-z.

## Introduction

Sex differences in migraine frequency, intensity and clinical presentation are well documented, with women affected two to three times more often and experiencing longer-lasting and more severe attacks [1, 2]. Although the underlying causes are multifactorial, fluctuations in sex hormones play a significant role in modulating migraine activity [3]. Historically, research has focused primarily on estrogen, with seminal work by Somerville et al. shaping the “estrogen withdrawal hypothesis” [4]. This theory posits that a rapid decline in estrogen levels, such as that occurring in the late luteal phase before menstruation, may act as a trigger for migraine attacks [4]. Given the complexity of hormonal dynamics throughout the menstrual cycle, it appears likely that estrogen alone does not account for the hormonal modulation of migraine. As a result, attention has turned to additional hormonal mediators, in particular oxytocin [5, 6]. Synthesized in the hypothalamus, oxytocin acts through the oxytocin receptor (OTR) and is involved in a wide range of physiological processes, including labor facilitation, social bonding, modulation of stress and pain regulation [7]. Similar to estrogen, oxytocin follows a cyclical pattern in women: levels decline prior to menstruation, rise and remain elevated throughout pregnancy, drop sharply in the postpartum period, and are consistently low after menopause [8]. Beyond its temporal correlation with phases of increased migraine susceptibility, further evidence suggests a potential role for oxytocin in the pathophysiology of migraine. As mainly derived from studies in rodents, but also highlighted by a few human postmortem tissue or autoradiography studies [9, 10], OTRs are expressed in brain regions implicated in migraine, including the trigeminal ganglion, a key neuroanatomical site in migraine initiation [11, 12]. Furthermore, according to animal models, there is growing evidence for an interaction between oxytocin and calcitonin gene-related peptide (CGRP), a neuropeptide considered pivotal in migraine development [5, 13]. In rodents, both OTRs and CGRP receptors are co-expressed in trigeminal ganglion neurons, and a preclinical study in rats demonstrated that oxytocin can suppress CGRP release [11]. In addition, recent findings from a rodent model has shown that hormones, in this case estrogen, can regulate CGRP and RAMP1 expression at the transcriptional level [14], so that oxytocin’s influence on migraine may extend beyond acute neuropeptide modulation and include slower, hormone-dependent gene regulatory mechanisms.

Another potential mechanism by which oxytocin could influence migraine involves its vascular properties. As shown in humans and animal models, oxytocin acts on receptors located on vascular endothelial and smooth muscle cells by which it can modulate vascular tone [15, 16]. Given that migraine attacks are associated with dynamic changes in meningeal and cerebral blood vessels [17], the role of oxytocin in vascular regulation could contribute to the modulation of migraine attacks.

Despite these converging lines of indirect evidence, a direct causal role of oxytocin in migraine pathophysiology in humans has yet to be demonstrated. Most studies have relied on correlational or preclinical data, leaving a critical gap in our understanding of the functional consequences of altered oxytocin signaling in humans. Given that migraine attacks in women are temporally associated with phases of low endogenous oxytocin levels, we hypothesized that reduced oxytocin signaling may increase migraine susceptibility. To explore this hypothesis, the present study investigated the effects of acute OTR blockade in women with migraine, compared to a healthy female control group and men with migraine. By pharmacologically inhibiting OTRs using the selective antagonist atosiban, we aimed to transiently suppress oxytocin signaling and assess its impact on headache induction and cerebrovascular dynamics.

## Methods

**Fig. 1:**
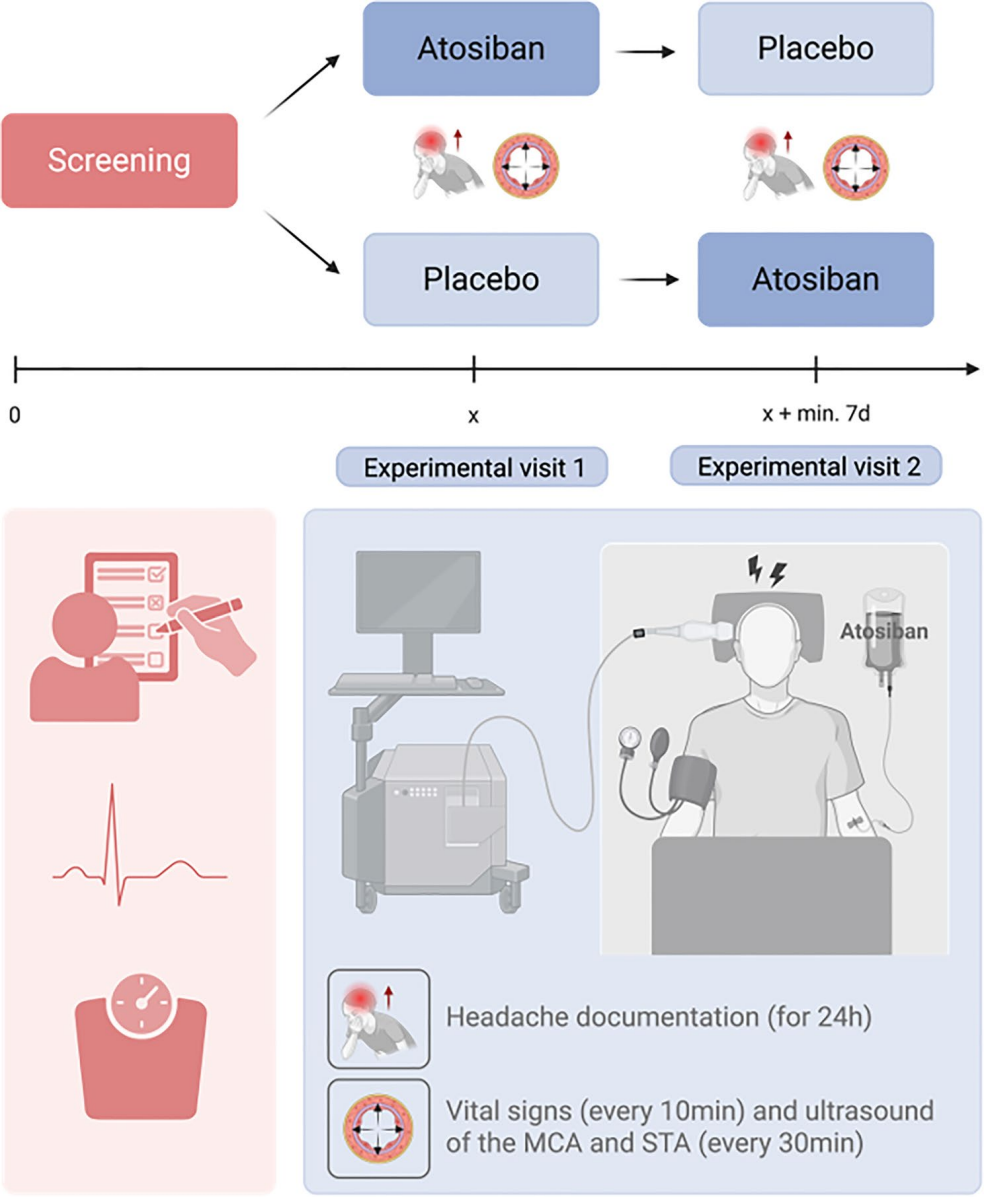
Study design. After a screening visit (red) to assess eligibility and baseline parameters, participants underwent two experimental visits (blue) in a randomized, double-blind, cross-over design. Each visit was preceded by ≥48-hour headache- and medication-free period. Participants received intravenous atosiban or placebo, followed by the alternate treatment at the second visit. During a 180-minute infusion, headache characteristics, vital signs, side effects (every 10 minutes), superficial temporal artery (STA) diameter, middle cerebral artery (MCA) blood flow velocity (every 30 minutes) were assessed. After discharge, participants completed hourly headache diaries for 21 hours. Created with BioRender.com

We conducted a single-center, double-blind, randomized, placebo-controlled human provocation study in a cross-over design using the OTR-antagonist atosiban (Tracotile^®^; Ferring Pharmaceuticals, Saint-Prex, Switzerland) as the provocation substance (Fig. 1).

The primary study group consisted of women with episodic migraine without aura who were recruited either directly through our tertiary headache center at Charité – Universitätsmedizin Berlin, via referrals from gynecologists in outpatient clinics, or through announcements at universities and other educational institutions. To assess whether migraine-specific or sex-specific pathophysiology influences the study outcomes, we included a control group of healthy women without migraine and a group of men with episodic migraine without aura. Eligible participants were aged 18–45 years. Women were required to use continuous hormonal contraception with amenorrhea for at least three months to minimize hormonal variablity. Migraine patients had to have a confirmed diagnosis of episodic migraine based on the criteria of the International Classification of Headache Disorders 3rd edition (ICHD-3) [18] and report between one and five migraine days in the 28 days prior to enrollment. Participants were excluded if they had a history of any (other) primary headache disorder, except for tension-type headache occurring no more than five days per month. Additional exclusion criteria for all groups were the presence of significant neurological, cardiovascular, psychiatric, metabolic, or inflammatory conditions; use of preventive migraine medication within 30 days or five plasma half-lives before enrollment (whichever longer); pregnancy, breastfeeding, plans to become pregnant; or a likelihood of non-compliance with the study protocol.

### Study visits

#### Screening visit

After confirming inclusion and exclusion criteria, we conducted a standardized semiquantitative interview and a physical examination, which included measurement of height, weight, blood pressure, and heart rate, a neurological examination, and a resting electrocardiogram. During the interview, we collected demographic data (sex, age, ethnicity), a detailed headache history, as well as relevant personal and family medical history (migraine in first-degree relatives).

#### Experimental visit

All experimental visits were conducted under standardized conditions, with participants being in a non-fasting and interictal state, defined as free from any headache or acute pain medication use for at least 48 h. Coffee, tea, cocoa, and other foods or beverages containing methylxanthines, as well as tobacco, were not permitted for at least 12 h prior to the experimental visit. If those conditions were not met, the session was rescheduled. Each participant received an intravenous infusion of either atosiban (6.75 mg bolus over 1 min, followed by continuous infusion of 54 mg/100 ml over 180 min) or placebo (0.9 ml NaCl bolus over 1 min, followed by continuous infusion of 100 ml over 180 min). Placebo matched atosiban in appearance, rate, and total volume to protect blinding. The dosage for atosiban corresponds to the saturation dosage recommended in the product information for the inhibition of preterm labor in obstetrics [19]. The provocation substance atosiban is a synthetic analogue resembling the nanopeptide structure of oxytocin acting as a competitive inhibitor of OTRs with a rapid onset of action and a terminal half-life of 1.7 h, making it well-suited for investigating immediate OTR blockade effects [20].

Subjects were given the alternate treatment during their second visit, with the order determined by a pre-generated randomization list. Both participants and staff involved in data collection remained blinded to treatment allocation. Infusions were prepared according to the randomization list by an unblinded study team that neither interacted with study participants nor took part in data analysis or interpretation. The randomization list was disclosed to the blinded investigators only after completion of both the study and the data analysis, which was performed by the blinded study team. The 180-minute infusion period was followed by a 20-minute observation phase in a controlled clinical setting. During the in-hospital phase, we monitored and collected headache occurrence and characteristics, vital signs, and adverse events at 10-minute intervals. In addition, sonographic assessments of the superficial temporal artery (STA) diameter and middle cerebral artery (MCA) blood flow velocity were conducted every 30 min (SonoScape E2, SonoScape Medical Corp., Shenzhen, China). Previous provocation studies have demonstrated that sonography and transcranial Doppler sonography (TCD) can detect changes in the diameter of the STA and cerebral blood flow velocity [21]. The MCA and STA were examined using two separate ultrasound probes at specific anatomical landmarks and the same side for each participant across both experiment days to ensure consistency. Within a single examination day, patients were marked with a water-soluble pen to guarantee the exact probe placement throughout the assessment. An average of four separate measurements were recorded for STA diameter at each time point (every 30 min from 0 to 180 min) forming the basis for calculating a corresponding mean STA value. After discharge, participants completed an hourly headache diary for a 21-hour follow-up period at home. Participants were permitted to treat occurring headaches with their usual acute migraine medication at any time. Intake and type of rescue medication was documented in the headache diary.

#### Endpoints

The primary preregistered endpoint of the study was the incidence of migraine-like attacks within a 12-hour observation period following administration of atosiban vs. placebo in women with migraine. Migraine-like attacks were defined in accordance with recommendations for provocation studies [22] as headache attacks that meet the criteria C and D of the ICHD-3 or headaches that resemble typical migraine attacks of the individual combined with the intake of acute medication (Table 1). Aggravation of headache by physical activity was documented by participants starting with the fourth hour post infusion, as participants were resting in the hospital during the infusion and observation phase. Secondary preregistered endpoints related to headache characteristics included the incidence of headache attacks (any kind) and headache intensity, both within the 12-hour observation period, following administration of atosiban vs. placebo in women with migraine, men with migraine and healthy controls. Headache intensity score was measured by a numerical rating scale (NRS), ranging from 0 (no pain) to 10 (worst imaginable pain). Additional secondary endpoints included the following vascular responses: mean arterial blood pressure (MAP), mean heart rate (beats per minute, BPM), STA diameter and MCA blood flow velocity following atosiban and placebo administration in women with migraine, men with migraine and healthy controls, assessed as the area under the curve (AUC) from baseline to 180 min.

As exploratory, non-preregistered endpoints, post hoc analyses were performed to evaluate migraine aura and to compare headache characteristics (including aura and headache intensity) across (a) women with migraine, (b) female controls, and (c) men with migraine within the 12 h observation period.

**Table 1:** Criteria for migraine-like attacks (primary endpoint)

Option 1	Option 2
Headache has at least two of the following four characteristics: 1. Unilateral location 2. Pulsating quality 3. Moderate or severe pain intensity 4. Aggravation by or causing avoidance of routine physical activity (e.g. walking or climbing stairs)	Headache mimics the usual migraine of the participant **AND** Headache is treated with acute migraine medication
**AND** During headache at least one of the following: 1. Nausea and/or vomiting 2. Photophobia and phonophobia	

### Statistical analyses

For statistical analysis we used IBM SPSS Statistics (IBM SPSS Statistics ©; 23.0, for Mac). As no prior studies had investigated migraine provocation rates using an OTR antagonist, the sample size calculation was based on data from substances known to reliably induce migraine attacks, specifically CGRP and PACAP, which exhibit average provocation rates of ≈ 60% [22]. The sample size calculation was performed for a one-sided McNemar’s test for the primary endpoint, assuming a significance level of 0.05 and a statistical power of 80%. Based on the assumption that placebo would provoke migraine-like attacks in approximately 10% of participants [23], and atosiban in 58%, a total of 20 women with migraine completing both experimental visits was determined to be adequate to detect a statistical difference. For both control groups, 20 healthy women and 20 men with migraine were recruited, which was deemed adequate to detect any unexpectedly high provocation responses in this populations. Data are reported according to their distribution as frequencies and percentages, means with standard deviations, or medians with interquartile ranges. The incidence of migraine and headache attacks under atosiban vs. placebo was analyzed using McNemar’s test for paired categorical data. AUC values for the headache intensity, MAP, mean heart rate, STA diameter and MCA blood flow velocity (AUC 0–180 min) after atosiban and placebo were compared using the Wilcoxon signed-rank test or paired-sample t-test, depending on the distribution of the data. Differences between groups (Female control group vs. Women with migraine; women with migraine vs. men with migraine) in the occurrence of (migraine) headaches were assessed using the chi-squared test. Differences in headache intensity were analyzed using either the Mann-Whitney U test or the independent-samples t-test, depending on whether the data were normally distributed.

## Results

**Fig. 2:**
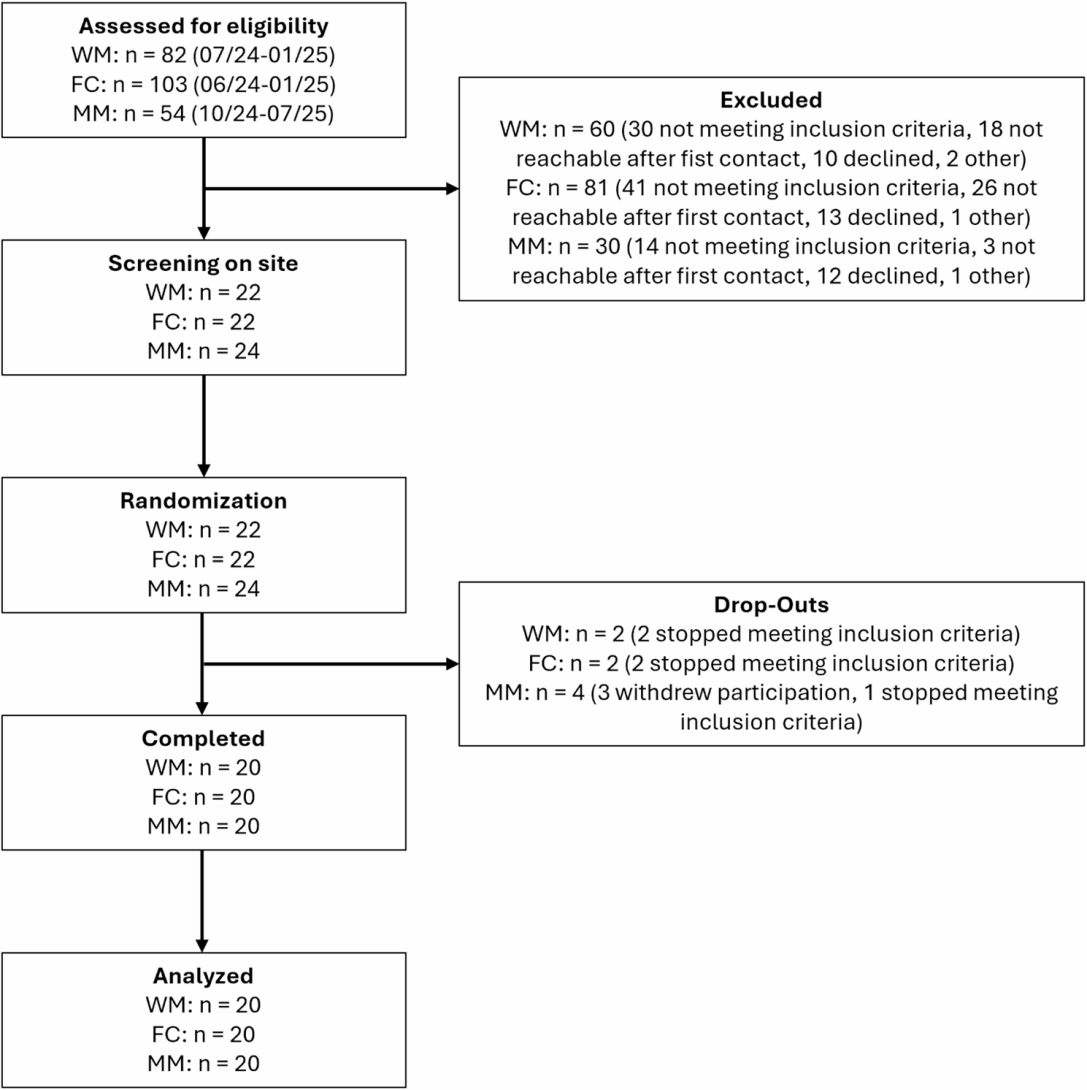
Participant flow. Abbr.: WM - Women with Migraine, FC - Female control group, MM - Men with Migraine

Between Februrary 2024 and July 2025, we screened a total of 239 potential participants, for details please refer to Fig. 2. Ultimately, 20 women with migraine, 20 healthy female controls, and 20 men with migraine completed the study. Among the 20 healthy female control participants, one reported a positive family history of migraine in a first-degree relative. Demographics are summarized in Table 2.

**Table 2:** Study population and headache characteristics

	Women with migraine *n* (%) or mean ± SD Total, 20 (100)	Female control group *n* (%) or mean ± SD Total, 20 (100)	Men with migraine *n* (%) or mean ± SD Total, 20 (100)
Female sex	20 (100)	20 (100)	0 (0)
Age (years)	27.1 ± 5.6	25.7 ± 6.8	29.3 ± 6.1
Ethnicity	White/European: 16 (80) Middle Eastern/North African: 4 (20)	White/European: 15 (75) Middle Eastern/North African: 3 (15) Black/African: 2 (10)	White/European: 17 (85) Middle Eastern/North African: 1 (5) Hispanic/Latino: 1 (5) Asian: 1 (5)
BMI (kg/m^2^)	21.3 ± 2.1	22.5 ± 3.2	23.7 ± 2.9
Contraception	COC: 4 (20) POP: 14 (70) Vaginal ring: 2 (10)	COC: 10 (50) POP: 8 (40) Vaginal ring: 2 (10)	-
Disease duration (years)	10.3 ± 7.0	-	15.3 ± 8.6
MMD	3.2 ± 1.3	-	3.1 ± 1.4
MHD	3.5 ± 1.8	1.5 ± 0.6	2.1 ± 1.8

Abbr. TTH – tension type headache, MHD – monthly headache days, MMD – monthly migraine days, COC – combined oral contraception, POP – Progestin-only pill

### Headache response after short-acting oxytocin-receptor blockage

#### Women with migraine

During the 12-hour observation period, six women with migraine (30%) experienced a migraine-like attack following atosiban compared to four (20%) after placebo (p = 0.75). No one experienced migraine-like attacks on both provocation days. Details of the individual headache attacks are provided in the Supplementary Table 1. Headaches of any kind were reported by 15 (75%) during the 12-h follow-up after atosiban and 11 (55%) after placebo (p = 0.34). Eight (40%) experienced headaches of any kind on both provocation days. Symptoms consistent with migraine aura occurred in two participants (10%) following atosiban, one with spreading sensory symptoms and one with combined sensory and visual symptoms. Both events occurred within the first 12 h. Similarly, after placebo, aura-like symptoms were reported by two participants (10%), including one case of visual symptoms and one case of combined sensory and visual symptoms.

#### Female control group

Female controls did not develop migraine-like attacks within the 12-hour observational period after atosiban nor placebo. Headaches of any kind occurred in nine participants (45%) within 12 h after atosiban, among whom was the participant with a positive family history of migraine and six (30%) participants after placebo (*p* = 0.25 for 12 h). Six participants (30%) experienced headaches of any kind on both provocation days. Symptoms consistent with migraine aura occurred in one participant (5%) after placebo, described as a scintillating scotoma. The AUC of headache intensity over 12 h did not differ significantly between the two treatment arms (AUC 0–12 h, *p* = 0.31).

#### Men with migraine

Among men with migraine, migraine-like attacks occurred within the 12-hour observational period in two participants (10%) after atosiban and in three participants (15%) after placebo (*p* > 0.999). No additional migraine-like attacks were observed within the subsequent 12-hour extended observation phase. No one experienced migraine-like attacks on both provocation days. Details of individual headache attacks are provided in the Supplementary Table 2. Headaches of any kind were reported in 11 participants (55%) within 12 h following atosiban, compared with 10 (50%) following placebo (*p* > 0.999). The integrated headache intensity over 12 h did not differ significantly between the two treatment arms (AUC 0–12 h, *p* = 0.71;Wilcoxon test). Aura-like symptoms occurred in three participants (15%) after atosiban provocation, two with visual symptoms and one with sensory symptoms, all within the first 12 h; no aura-like symptoms were reported after placebo.

#### Women with migraine vs. female control group

Women with migraine tended to experience more often headache of any kind within the 12-hour observation period following administration of atosiban compared to the female control group (15/20 vs. 9/20, *p* = 0.053) and had a significantly higher headache intensity (AUC 0–12 h, *p* = 0.043).

#### Women with migraine vs. men with migraine

**Fig. 3:**
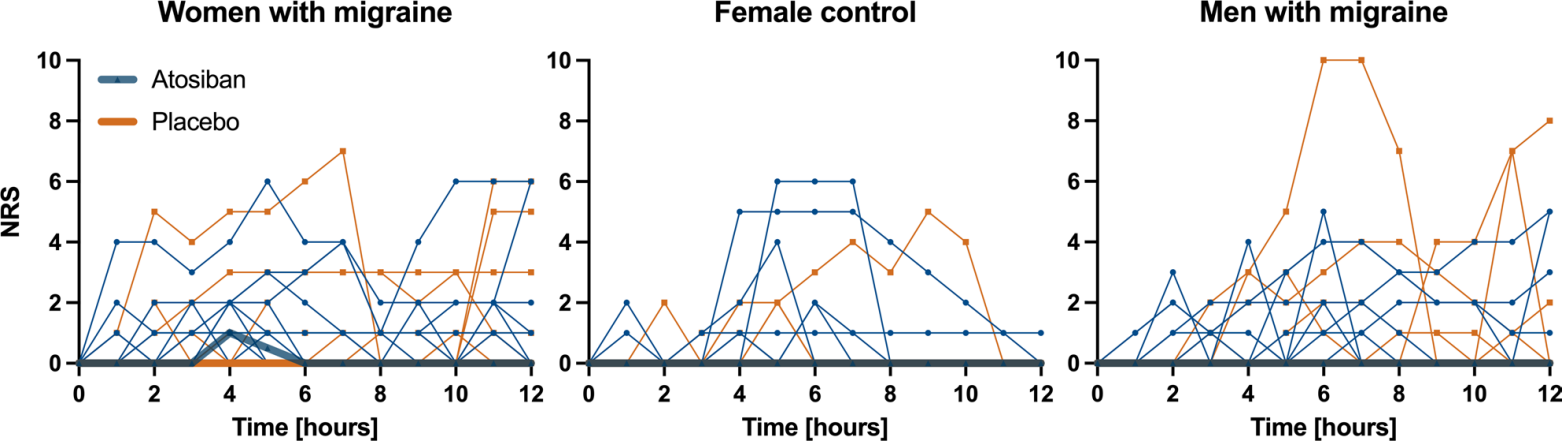
Pain intensity scores after atosiban or placebo administration. Median headache intensity score (thick lines) and individual participant headache scroes (thin lines) on the numerical rating scale (NRS) following atosiban (blue) or placebo (orange) in women with migraine (left), female control group (middle) and men with migraine (right)

The frequency of migraine-like attacks (6/20 vs. 2/20, *p* = 0.11) or headache of any kind (15/20 vs. 11/20, *p* = 0.19) did not differ significantly between women and men with migraine within 12 h after atosiban. Headache intensity did not differ significantly between sexes (AUC 0–12 h, *p* = 0.31) (Fig. 3).

### Vascular response after short-acting oxytocin-receptor blockage

#### Women with migraine

**Fig. 4:**
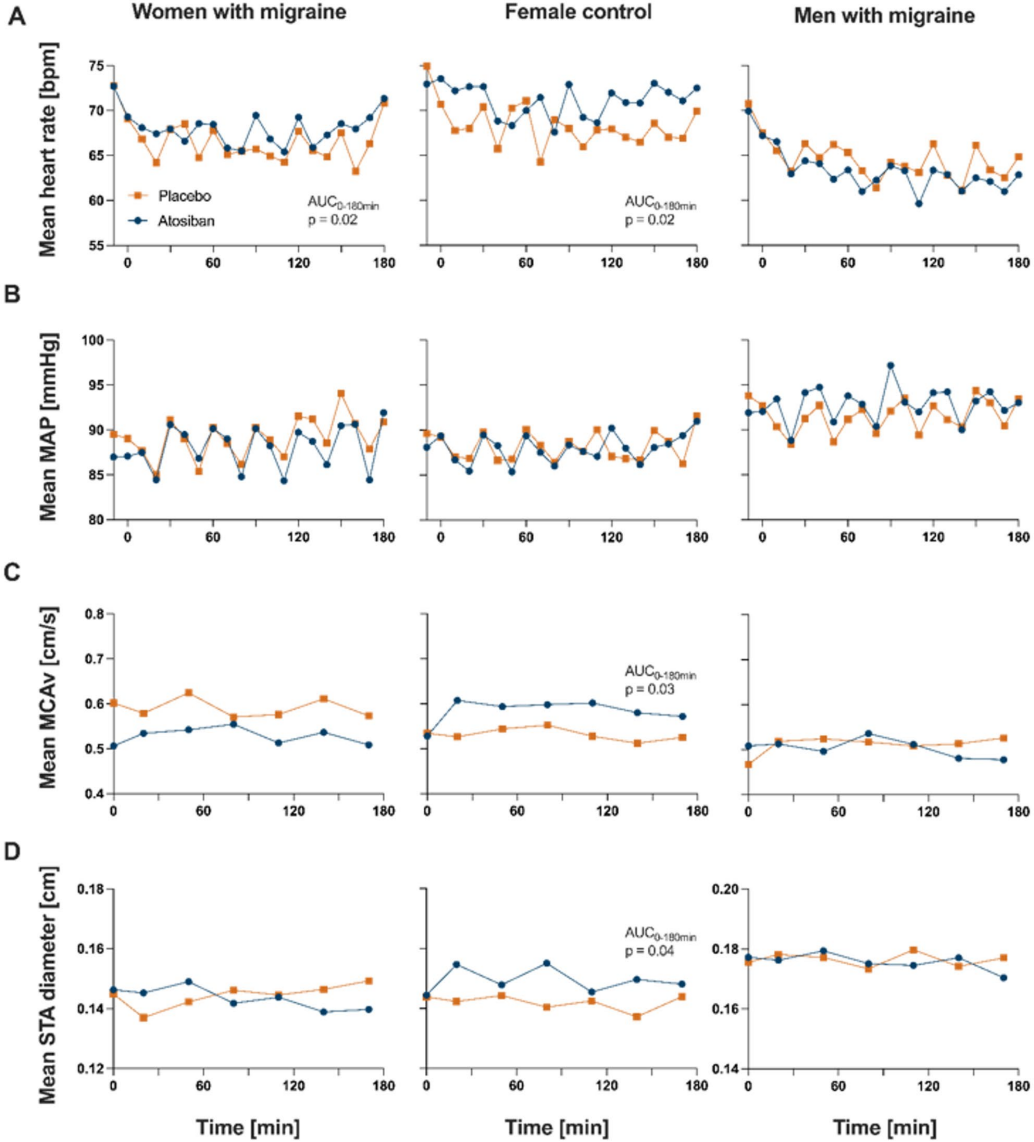
Vascular parameters after atosiban or placebo administration. Mean heart rate (**A**), mean arterial blood pressure (**B**), mean blood velocity of the middle cerebral artery (MCA) (**C**) and mean diameter of the superficial temporal artery (STA) (**D**) during the 180 min after atosiban (blue) or placebo (orange) administration in women with migraine (left), female control group (middle) and men with migraine (right)

During the 180-minutes monitoring phase following atosiban administration, no significant differences were observed in MAP (AUC 0–180 min, *p* = 0.43) compared to placebo in women with migraine (Fig. 4). Following atosiban, mean heart rate was significantly higher compared to placebo (AUC 0–180 min: 1202.3 vs. 1172.0, *p* = 0.02). We did not identify significant differences in the diameter of the STA (AUC 0–180 min, *p* = 0.85) or in MCA flow velocity (AUC 0–180 min, *p* = 0.19).

#### Female control group

In healthy female controls, no difference in MAP was observed following 180 min after administrating atosiban or placebo (AUC 0–180 min, *p* = 0.99). However, we identified a significant difference in the AUC of the mean heart rate over 180 min between atosiban and placebo conditions, with a higher heart rate after atosiban (median AUC 0–180 min: 1235.3 vs. 1215.0; *p* = 0.02) in parallel with a significantly increased AUC of the STA under atosiban compared to placebo (AUC 0–180 min: 0.90 vs. 0.85; *p* = 0.04). Furthermore, the AUC for the MCA blood flow velocity was significantly higher following atosiban compared to placebo (median AUC 0–180 min: 3.57 vs. 3.29; *p* = 0.03).

#### Men with migraine

No significant differences were observed within the 180-minutes observation period after atosiban in MAP (AUC 0–180 min, *p* = 0.30) or mean heart rate (AUC 0–180 min, *p* = 0.48) compared with placebo (Fig. 4). In addition, no significant differences were detected in STA diameter (AUC 0–180 min, *p* = 0.94) or MCA flow velocity (*p* = 0.93).

#### Adverse events

In total, nine women with migraine (45%), four female healthy controls (20%) and five men with migraine (25%) reported adverse events in the 12-hour observational period after administration of atosiban. This was in eight cases feeling of warmth (8/18, 44%) followed by dizziness (6/18, 33%). After placebo administration, seven women with migraine (35%), three female healthy controls (15%) and four men with migraine (20%) reported adverse events, which was in four cases (4/14, 27%) feeling of warmth and in three cases (3/14, 21%) palpitations. Details are provided in the supplements (Supplementary Tables 3–5).

## Discussion

In this double-blind, placebo-controlled human pilot provocation study, antagonism of the OTR using atosiban did not lead to an increased incidence of migraine-like attacks in women with migraine when compared to placebo. This finding was consistent across sexes and also extended to the overall headache frequency. It should be noted, however, that a high placebo response was observed, which may have limited the sensitivity to detect small or moderate drug-specific effects and should be considered when interpreting the results. Nevertheless, several pathophysiological and pharmacological mechanisms may account for these findings.

### Limited role of isolated oxytocin signaling in initiating migraine attacks

Reduced oxytocin signaling alone may be insufficient to initiate migraine attacks. Migraine pathophysiology is influenced by the complex interplay of several different hormones [5]. In particular, cyclic changes in sex hormones such as estrogen and progesterone are closely associated with migraine occurrence [24]. As shown mostly in animal models but also humans, estrogen and other sex hormones exert significant regulatory effects on the oxytocin system including the synthesis, release, and receptor expression [13, 25, 26]. In preclinical in vivo studies estrogen has been shown to stimulate oxytocin mRNA expression and release from the hypothalamus [12, 27, 28]. Observations from a prospective cohort pilot study revealed positive correlations between serum oxytocin and estradiol and negative correlations with progesterone, which could suggest that estrogen and progesterone exert opposing regulatory effects on oxytocin levels in humans [26]. Moreover, in animal models estrogen stimulates OTR binding capacity in various brain regions by enhancing OTR mRNA expression [29]. Evidence from animal studies as well as research on human tissues further indicate that estrogen receptors are widely distributed in migraine-relevant brain areas, including many hypothalamic nuclei that also secrete oxytocin [13, 30], and are found in substantial proportions on human as well as rat oxytocinergic neuronal populations [31]. In addition, estrogen is thought to regulate other migraine-related mediators such as CGRP [30, 32]. This dual regulatory role may indicate that migraine susceptibility is influenced by a balance between pro-nociceptive factors (e.g., CGRP) and anti-nociceptive factors, with oxytocin potentially serving as an anti-nociceptive mediator [13]. Hormonal fluctuations, such as those during the menstrual cycle, may disrupt this balance and increase vulnerability to attacks. In this framework, oxytocin may act as a modulatory factor that shapes the overall threshold for migraine susceptibility, but the transient absence of oxytocin is insufficient on its own to act as a primary trigger. Although the use of continuous hormonal contraception allowed us to minimize the influence of naturally fluctuating hormone concentrations, particularly estrogen, on the study outcome it may nevertheless have attenuated the effect of the oxytocin receptor antagonist. As discussed above, in rodents, higher estrogen levels are associated with increased oxytocin receptor density. Under continuous hormonal contraception, both estrogen and oxytocin levels remain relatively low and stable, which may in turn influenced oxytocin receptor expression or density. This hormonal milieu could have resulted in decreased receptor responsiveness and, consequently, a reduced sensitivity to treatment with the oxytocin receptor antagonist. It should be noted that most studies investigating the relationship between oxytocin, estrogen, other sex hormones and the CGRP signaling system are based on preclinical research in rodents, and therefore extrapolation to humans, and any causal inference, must be made with great caution.

Methodological factors may also have contributed to the absence of an effect in our study. While oxytocin withdrawal in the perimenstrual period lasts for several days [8], the experimentally induced blockade of OTR in our study was limited to about three hours, which may not have been long enough to mimic the in vivo hormonal conditions that facilitate migraine initiation. Administration of atosiban over several days would undoubtedly be scientifically intriguing; however, it is neither practically feasible nor ethically justifiable.

Interestingly, 15% of men with migraine and 10% of women with migraine experienced aura-like symptoms after provocation with atosiban. To the best of our knowledge, there is no direct evidence linking oxytocin to the induction of spreading depolarization, the underlying pathophysiological correlate of migraine aura [33]. However, the OTR antagonist L-368,899 was shown to dose-dependently prolong SD-induced periorbital allodynia in rodents [34]. Given that none of the patients had a prior history of migraine with aura, this finding warrants further investigation in future studies that are adequately powered to explore this phenomenon. However, two women with migraine but also one healthy female control participants reported aura-like symptoms after placebo administration, raising the possibility that some of these events may reflect nocebo responses rather than true pharmacological effects. Further, it should be noted that this is an exploratory outcome, and the study was not powered to detect effects for this measure.

### Limited central availability of atosiban

Another potential explanation for our findings concerns the way oxytocin acts in migraine and how this may or may not be influenced by atosiban, depending on its ability to reach central targets.

Oxytocin is secreted mainly by the paraventricular and supraoptic nuclei of the hypothalamus [35] and robustly projects to key regions involved in migraine pain processing, including the trigeminocervical complex (TCC) in rodents [36, 37]. Within these circuits, oxytocin reduces trigeminal excitability through GABAergic pathways, inhibits A-delta and C-fiber nociceptive transmission, suppresses CGRP release and thereby raises nociceptive thresholds [38–40]. In rodents and humans, OTRs are also expressed in key trigeminal structures, including the trigeminal ganglion, trigeminal nucleus caudalis, and the TCC [10, 11]. Importantly, with the exception of the trigeminal ganglion, these regions lie within the blood–brain barrier (BBB). Thus, the evidence suggests that oxytocin’s contribution to migraine could be largely mediated through central neural mechanisms.

Against this background, it remains an open question whether atosiban can effectively interact with central oxytocinergic pathways. Given its molecular weight and hydrophilic properties, atosiban likely has limited BBB penetration under normal physiological conditions. Nevertheless, some rodent studies suggest that systemic administration may induce central effects [41], such that analogous central activity in humans cannot be definitively excluded. Whether comparable penetration occurs in humans, however, remains uncertain. Consequently, the present findings may primarily reflect peripheral OTR antagonism and may not adequately capture the effects of sustained central oxytocin blockade. Taken together, these considerations suggest that investigating OTR antagonism in migraine may require longer-acting and reliably brain-penetrant compounds, as well as study designs allowing prolonged exposure over days to weeks, in order to assess potential cumulative effects on headache frequency and severity.

### Altered vascular responsiveness in migraine patients

Oxytocin exerts complex and context-dependent vascular effects. In the human cerebrovascular system, OTRs are expressed in the walls of intracranial arteries such as the common carotid artery and the middle meningeal artery, where they are located exclusively on vascular smooth muscle cells [16]. In this setting, oxytocin leads to a moderate vasoconstriction, second in potency to vasopressin [16]. In the peripheral circulation, oxytocin likewise induces vasoconstriction in a dose dependent way, likely mediated via vasopressin V1A receptors rather than OTR [42, 43]. In line with these vascular effects, in our study OTR blockade in healthy controls was associated with a dilation of the STA compared to placebo, an effect that was absent in both women and men with migraine. In healthy participants, administration of atosiban was further found to be associated with an increased heart rate and elevated flow velocity in the MCA. With the exception of a significant increase in heart rate in women with migraine, these effects were largely absent in both analyzed migraine cohorts. The physiology underlying the findings in healthy subjects remain elusive at this point but could be understood in the context of oxytocin’s well-described cardiovascular actions. In animal models oxytocin was shown to exert negative inotropic and chronotropic actions, mediated through OTR expressed in cardiac tissue, largely via pathways involving intrinsic cardiac cholinergic neurons and NO signaling [44]. In humans, receptor activation reduces heart rate and contractility, lowers afterload through vasodilatation, and contributes to reduced blood pressure [45]. Blockade of these receptors consequently removes oxytocin’s parasympathetic neuromodulatory influence, resulting in an increase in heart rate and enhanced myocardial contractility [15]. Since cardiac function and anatomy are generally preserved in patients with migraine, this may help to explain why an increase in heart rate was the only vascular parameter that rose significantly in women with migraine under atosiban compared to placebo. Along with the rise in heart rate, healthy women exhibited increased MCA flow velocity under atosiban compared with placebo. This appears to be a plausible consequence of a concomitant increase in cardiac output leading to a greater cerebral blood flow [46]. Overall, interpreting the vascular data is challenging and somewhat contradictory, as increased MCA flow velocity could also suggest vasoconstriction, a phenomenon that was not detected in the study. In addition, the study was not powered to evaluate vascular parameters, which should therefore be interpreted as exploratory and hypothesis-generating rather than confirmatory.

Importantly, in healthy vessels, both vascular smooth muscle cells and endothelial cells maintain intact contractile and regulatory function, enabling dynamic modulation of vessel tone in response to neurohumoral stimuli. In subjects with migraine, by contrast, such vascular adaptability appears to be compromised, which may explain why atosiban failed to elicit comparable vascular effects in this group. A growing body of evidence supports the notion of altered vascular responsiveness in migraine and structural changes within the vessel wall [47–50]. These observations point toward a disease-specific limitation in vascular adaptability among migraine patients. Altered vascular structure and function may attenuate the effects of OTR blockade, thereby preventing atosiban from producing measurable hemodynamic or clinical effects in this population.

### Strengths and limitations

A key strength of this study lies in the rigorous methodological design. The placebo-controlled, double-blind approach strengthens internal validity. Several limitations should be acknowledged. First, the sample size was calculated based on provocation rates of CGRP as the provocations substance, since no prior data were available for oxytocin when this study started. Nevertheless, the magnitude of the placebo response was in line with what has been reported in other provocation studies [23], supporting the reliability of our data. However, the still substantial placebo response may have reduced the ability to detect small treatment-related effects. Potential explanations for this pronounced placebo effect include (1) the highly controlled experimental environment with close monitoring, which can enhance expectancy and symptom awareness, (2) anticipatory anxiety or heightened attentiveness to bodily sensations, which could promote headache reporting independent of the intervention and (3) the crossover design, which may amplify placebo responses through repeated exposure to study procedures and increased familiarity with perceived sensations. Importantly, the study was powered for the cohort of women with migraine. The secondary endpoint analyses including men with migraine and healthy control participants were therefore of an exploratory nature and should be interpreted with appropriate caution. Second, although migraine-like attacks were defined according to established criteria [22], aggravation by physical activity was not systematically tested during the 4-hour in-hospital phase (e.g., by instructed coughing or neck movement to assess pain exacerbation), which differs from procedures applied in other provocation studies. Third, atosiban is a peptide antagonist with high affinity not only for the oxytocin receptor but also for the vasopressin V1a receptor [51]. Consequently, the observed vascular effects may not be attributable solely to OTR blockade. Fourth, circulating hormone concentrations were not measured. Based on previous evidence, we assumed stable hormone levels under continuous hormonal contraception [52, 53]; however, both women using progestin-only regimens and those on combined oral contraceptives were included, which may have introduced heterogeneity. Furthermore, as all female participants were required to use continuous hormonal contraception, our findings may not be generalizable to women experiencing natural menstrual cycles with physiological hormone fluctuations. Finally, both the provocation substance and the placebo triggered headaches in a substantial number of cases, suggesting that expectancy effects may have contributed.

## Conclusion

Short-acting antagonism of the OTR using atosiban did not induce migraine-like attacks in individuals with migraine. This finding suggests that transient and acute suppression of oxytocin signaling alone is unlikely to provoke migraine attacks under stable hormonal conditions. Considering the neuromodulatory role of oxytocin and its interaction with complex hormonal and neurovascular mechanisms, at least short-acting OTR antagonism may be insufficient to substantially affect migraine pathophysiology. Instead, the reciprocal modulatory interactions with oxytocin and other hormonal systems and neurovascular mechanisms, likely occurring over longer timescales, may be of greater relevance. Future studies might therefore benefit, where ethically feasible, from investigating longer-lasting OTR blockade or compounds with a reliable blood–brain barrier penetration under physiological hormonal conditions but also the direct effects of oxytocin administration to more comprehensively clarify the role of oxytocin and its antagonism in migraine susceptibility. 

## Supplementary Information

Below is the link to the electronic supplementary material.

Supplementary Material 1

## Data Availability

The datasets used and/or analyzed during the current study are available from the corresponding author on reasonable request.

## Abbreviations

AUC: Area under the curve
BBB: Blood–brain barrier
BPM: Beats per minute
CGRP: Calcitonin gene–related peptide
ICHD: International classification of headache disorders
MAP: Mean arterial blood pressure
MCA: Middle cerebral artery
NO: Nitric oxide
NRS: Numeric rating scale
OTR: Oxytocin receptor
STA: Superficial temporal artery
TCC: Trigeminocervical complex
