# Supplementary material for: Oxytocin receptor antagonism in migraine: a randomized, double-blind, placebo-controlled provocation study
Source: J Headache Pain. 2026 Feb 16;27(1):54. doi: 10.1186/s10194-026-02297-z (PMC12927216; doi:10.1186/s10194-026-02297-z)
Supplement: Supplementary file 1 — Supplementary Material 1 [file 10194_2026_2297_MOESM1_ESM.docx]

**Supplemental Table 1. Characteristics of provoked headache in women with migraine (0-12 hour observation period)**

| **ID** |  | **Peak headache (duration of headache)** | **Headache characteristics^a^** | **Associated symptoms^b^** | **Migraine aura^c^** | **Mimics usual migraine** | **Migraine-like attack (onset)** | **Treatment (time)** |  |
| --- | --- | --- | --- | --- | --- | --- | --- | --- | --- |
| **113** | Spontaneous | (> 48h) | Unilat/3/puls/+ | +/+/+ | -/- | - | - | - |  |
|  | Atosiban (1) | 5h (3h) | Unilat/1/press/- | -/-/- | -/- | No | na | No |  |
|  | Placebo | 5h (9h20min) | Bilat/6/press/+ | +/+/+ | -/- | Yes | 5h | Yes (5h, 11h) |  |
| **114** | Spontaneous | (6h) | Unilat/4/puls/+ | +/+/+ | -/- | - | - | - |  |
|  | Atosiban (2) | None |  |  |  |  |  |  |  |
|  | Placebo | None |  |  | +/- |  |  |  |  |
| **115** | Spontaneous | (6-8h) | Bilat/6/puls, press/+ | +/+/- | -/- | - | - | - |  |
|  | Atosiban (1) | 5h (11h30min) | Unilat/6/puls/+ | +/+/+ | -/- | Yes | 2h | Yes (5h,12h) |  |
|  | Placebo | None |  |  |  |  |  |  |  |
| **116** | Spontaneous | (48h) | Bilat/7/puls, press/+ | +/+/+ | -/- | - | - | - |  |
|  | Atosiban (1) | 3h (2h) | Bilat/1/press/+ | -/-/- | -/- | Yes | na | No |  |
|  | Placebo | None |  |  |  |  |  |  |  |
| **117** | Spontaneous | (24h) | Bilat/6-7/puls/+ | +/+/- | -/- | - | - | - |  |
|  | Atosiban (1) | 100min (10min) | Bilat/3/puls/- | -/-/- | -/- | No | na | No |  |
|  | Placebo | 11h (2h) | Unilat/5/puls/+ | +/-/- | -/- | No | na | Yes (11h) |  |
| **119** | Spontaneous | (5h)^d^ | Unilat/7/puls/+ | +/+/- | -/- | - | - | - |  |
|  | Atosiban (2) | None |  |  |  |  |  |  |  |
|  | Placebo | None |  |  |  |  |  |  |  |
| **120** | Spontaneous | (24h) | Bilat/6/puls/+ | +/+/+ | -/- | - | - | - |  |
|  | Atosiban (2) | None |  |  |  |  |  |  |  |
|  | Placebo | 6h (8h) | Bilat/3/puls/+ | -/-/+ | -/- | No | 6h | No |  |
| **121** | Spontaneous | (0,5-6h) | Bilat/6/puls, press, burn/- | +/+/+ | -/- | - | - | - |  |
|  | Atosiban (1) | 150min (5h10min) | Unilat/3/press/+ | +/+/+ | +/+ | Yes | 4h | No |  |
|  | Placebo | 10h (2h) | Unilat/1/press/- | -/+/+ | +/+ | No | na | No |  |
| **123** | Spontaneous | 12h | Bilat/6/puls, press, burn/+ | +/+/+ | -/- | - | - | - |  |
|  | Atosiban (2) | None |  |  |  |  |  |  |  |
|  | Placebo | 7h (10h20min) | Unilat/7/puls/+ | +/+/- | -/- | Yes | 120min | Yes (120min, 6h) |  |
| **124** | Spontaneous | (8h) | Bilat/7/puls/+ | +/+/+ | -/- | - | - | - |  |
|  | Atosiban (1) | 10h (10h50min) | Unilat/2/press/- | -/-/- | -/- | No | na | No |  |
|  | Placebo | 4h (11h) | Unilat/3/press/+ | +/-/- | -/- | Yes | na | No |  |
| **125** | Spontaneous | (8h) | Bilat/7/puls/+ | +/+/- | -/- | - | - | - |  |
|  | Atosiban (2) | 11h (1h) | Bilat/1/press/+ | +/-/- | -/- | No | na | No |  |
|  | Placebo | 7h (1h) | Bilat/1/press/+ | -/-/+ | -/- | No | na | No |  |
| **126** | Spontaneous | (24-48h) | Bilat/7/puls/+ | +/+/+ | -/- | - | - | - |  |
|  | Atosiban (1) | 7h (5h) | Unilat/4/puls/+ | +/+/- | -/- | Yes | 6h | Yes (7h) |  |
|  | Placebo | 8h (3h) | Bilat/1/press/- | -/-/- | -/- | No | na | No |  |
| **127** | Spontaneous | (24h) | Bilat/7/puls/+ | +/+/+ | -/- | - | - | - |  |
|  | Atosiban (1) | 50min (7h30min) | Unilat/1/puls/+ | -/-/+ | -/- | Yes | 4h | No |  |
|  | Placebo | None |  |  |  |  |  |  |  |
| **128** | Spontaneous | (8h) | Bilat/8/puls/+ | +/+/+ | -/- | - | - | - |  |
|  | Atosiban (2) | None |  |  |  |  |  |  |  |
|  | Placebo | 110min (1h) | Unilat/3/press/^e^ | -/-/+ | -/- | No | na | No |  |
| **129** | Spontaneous | (48h) | Unilat/7/puls/+ | +/+/- | -/- | - | - | - |  |
|  | Atosiban (2) | 10h (10h40min) | Bilat/6/press/- | +/-/- | -/- | Yes | na | No |  |
|  | Placebo | 11h (2h) | Bilat/6/press/+ | +/-/- | -/- | Yes | 11h | Yes (11h) |  |
| **130** | Spontaneous | (72h) | Bilat/6/puls/+ | +/+/+ | -/- | - | - | - |  |
|  | Atosiban (1) | 4h (4h) | Unilat/2/press/- | +/-/- | -/+ | Yes | na | No |  |
|  | Placebo | None |  |  |  |  |  |  |  |
| **131** | Spontaneous | (6h) | Bilat/6/puls/+ | +/-/+ | -/- | - | - | - |  |
|  | Atosiban (2) | 4h (2h) | Unilat/2/puls/- | +/-/+ | -/- | Yes | 4h | No |  |
|  | Placebo | None |  |  |  |  |  |  |  |
| **132** | Spontaneous | (6h) | Bilat/7/puls/+ | +/+/- | -/- | - | - | - |  |
|  | Atosiban (1) | 5h (5h) | Unilat/3/press/- | +/-/- | -/- | No | Na | Yes (4h) |  |
|  | Placebo | None |  |  |  |  |  |  |  |
| **133** | Spontaneous | (48-72h) | Unilat/5/puls/+ | +/+/+ | -/- | - | - | - |  |
|  | Atosiban (1) | 7h (10h 30min) | Unilat/4/press/+ | -/+/- | -/- | Yes | 7h | Yes (7h) |  |
|  | Placebo | 4h (8h) | Bilat/1/press/- | -/-/- | -/- | No | na | No |  |
| **134** | Spontaneous | (24h) | Unilat/7/puls, press/+ | +/+/+ | - | - | - | - |  |
|  | Atosiban (2) | 9h (10h 40min) | Unilat/5/puls/+ | +/+/+ | -/- | Yes | 5h | Yes (9h) |  |
|  | Placebo | None |  |  |  |  |  |  |  |

^a^Localization (bilat=bilateral; unilat=unilateral)/intensity/quality (puls=pulsing; press=presssing; burn=burning)/aggravation by physical activity.

^b^Photophobia/phonophobia/nausea

^c^visual/sensoric

^d^unmedicated duration not known, time of medication intake

^e^Assessment initiated 4h post infusion

na – not applicable

**Supplemental Table 2. Characteristics of provoked headache in men with migraine(0-12 hour observation period)**

| **ID** |  | **Peak headache (duration of headache)** | **Headache characteristics^a^** | **Associated symptoms^b^** | **Migraine aura^c^** | **Mimics usual migraine** | **Migraine-like attack (onset)** | **Treatment (time)** | |  |
| --- | --- | --- | --- | --- | --- | --- | --- | --- | --- | --- |
|  | Spontaneous | (8h) | Bilat/5/puls/+ | +/+/+ | -/- | - | - | - | |  |
| **201** | Atosiban (1) | 180min (1h) | Unilat/1/press/+ | -/-/+ | -/- | No | 180min | No | |  |
|  | Placebo | 6h (6h) | Bilat/2/puls/+ | -/-/- | -/- | Yes | na | Yes (9h) | |  |
|  | Spontaneous | (20h) | Unilat/4/puls, press/+ | +/+/- | -/- | - | - | - | |  |
| **202** | Atosiban (1) | 100min(3h20min) | Bilat/2/puls/- | +/-/- | +/- | Yes | na | No | |  |
|  | Placebo | None |  |  |  |  |  |  | |  |
|  | Spontaneous | (24-48h) | Bilat/6/press/- | +/+/- | -/- | - | - | - | |  |
| **203** | Atosiban (2) | None |  |  |  |  |  |  | |  |
|  | Placebo | None |  |  |  |  |  |  | |  |
|  | Spontaneous | (7h) | Unilat/5/press/+ | +/+/- | -/- | - | - | - |  |  |
| **204** | Atosiban (2) | None |  |  |  |  |  |  |  |  |
|  | Placebo | None |  |  |  |  |  |  | |  |
|  | Spontaneous | (6h) | Unilat/5/puls/+ | +/+/+ | -/- | - | - | - | |  |
| **205** | Atosiban (1) | 6h (6h) | Unilat/4/press/- | -/+/- | -/- | Yes | na | No | |  |
|  | Placebo | None |  |  |  |  |  |  | |  |
| **207** | Spontaneous | (12h) | Unilat/6,5/puls, press/+ | +/+/+ | -/- | - | - | - | |  |
|  | Atosiban (2) | 50min (30min) | Unilat/3/puls/^d^ | -/-/- | +/- | No | Na | No | |  |
|  | Placebo | 12h (4h) | Unilat/8/puls/+ | +/+/- | -/- | Yes | 9h | Yes (10h) | |  |
|  | Spontaneous | (5h) | Unilat/6/puls/+ | +/+/+ | -/- | - | - | - | |  |
| **208** | Atosiban (2) | None |  |  |  |  |  |  | |  |
|  | Placebo | None |  |  |  |  |  |  | |  |
|  | Spontaneous | (4-5h) | Bilat/4/puls/+ | +/+/+ | -/- | - | - | - | |  |
| **209** | Atosiban (1) | 7h (4h10min) | Unilat/2/press/+ | -/-/+ | -/- | Yes | na | No | |  |
|  | Placebo | 180min (2h10min) | Unilat/1/press/- | -/-/- | -/- | No | na | Yes (8h) | |  |
|  | Spontaneous | (24h) | Unilat/7/puls/+ | +/+/- | -/- | - | - | - | |  |
| **210** | Atosiban (1) | 12h (8h40min) | Unilat/3/puls/+ | +/-/- | -/- | No | na | Yes (13h) | |  |
|  | Placebo | None |  |  |  |  |  |  | |  |
|  | Spontaneous | (72h) | Unilat/8/puls, press/+ | +/+/+ | -/- | - | - | - | |  |
| **211** | Atosiban (2) | None |  |  |  |  |  |  | |  |
|  | Placebo | 6h (5h) | Bilat/10/press/+ | +/+/- | -/- | No | na | Yes (4h) | |  |
|  | Spontaneous | (12h) | Bilat/7/press/+ | +/+/- | -/- | - | - | - | |  |
| **212** | Atosiban (2) | 6h (2h10min) | Unilat/5/press/- | +/-/- | -/- | No | na | No | |  |
|  | Placebo | 11h (1h) | Bilat/7/press/- | +/-/- | -/- | No | na | No | |  |
|  | Spontaneous | (15h) | Unilat/6/puls/+ | +/+/+ | -/- | - | - | - | |  |
| **213** | Atosiban (2) | None |  |  |  |  |  |  | |  |
|  | Placebo | 5h (3h10min) | Unilat/3/press/+ | -/-/+ | -/- | Yes | 4h | Yes (5h) | |  |
|  | Spontaneous | (12h) | Bilat/6/press/+ | -/+/+ | -/- | - | - | - | |  |
| **214** | Atosiban (2) | 12h (11h30min) | Unilat/5/press/- | -/-/- | -/- | No | na | No | |  |
|  | Placebo | 180min (10min) | Bilat/1/press/^d^ | -/-/- | -/- | No | na | No | |  |
|  | Spontaneous | (5h) | Unilat/9/puls/+ | +/+/- | -/- | - | - | - | |  |
| **215** | Atosiban (1) | None |  |  |  |  |  |  | |  |
|  | Placebo | None |  |  |  |  |  |  | |  |
|  | Spontaneous | (24-48h) | Bilat/6/puls, press/+ | +/+/+ | -/- | - | - | - | |  |
| **216** | Atosiban (1) | 4h (3h40min) | Bilat/4/puls/- | -/+/+ | -/+ | Yes | na | No | |  |
|  | Placebo | None |  |  |  |  |  |  | |  |
|  | Spontaneous | (>4h) | Bilat/7/puls, press/+ | +/+/+ | -/- | - | - | - | |  |
| **217** | Atosiban (1) | None |  | -/-/+ |  |  |  |  | |  |
|  | Placebo | 100min (10min) | Bilat/1/press/^d^ | -/-/- | -/- | No | na | No | |  |
|  | Spontaneous | (16h) | Bilat/7/puls/+ | +/+/+ | -/- | - | - | - | |  |
| **219** | Atosiban (2) | None |  |  |  |  |  |  | |  |
|  | Placebo | None |  |  |  |  |  |  | |  |
|  | Spontaneous | (7h) | Unilat/6/puls, press/+ | +/+/+ | -/- | - | - | - | |  |
| **222** | Atosiban (2) | 7h (1h) | Unilat/1/press/- | -/-/- | -/- | Yes | 7h | Yes (7h) | |  |
|  | Placebo | 12h (2h) | Unilat/2/press/+ | +/+/- | -/- | Yes | 12h | No | |  |
|  | Spontaneous | (24h) | Unilat/5/puls, press/+ | +/+/+ | -/- | - | - | - | |  |
| **223** | Atosiban (2) | None |  |  |  |  |  |  | |  |
|  | Placebo | None |  |  |  |  |  |  | |  |
| **224** | Spontaneous | (10h) | Bilat/6/puls, press/+ | +/+/+ | -/- | - | - | - | |  |
|  | Atosiban (1) | 6h (9h50min) | Bilat/4/press/+ | +/+/+ | -/- | Yes | 6h | No | |  |
|  | Placebo | 7h (8h50min) | Bilat/4/puls/+ | +/-/- | -/- | No | na | Yes (7h) | |  |

^a^Localization (bilat=bilateral; unilat=unilateral)/intensity/quality (puls=pulsing; press=pressing; burn=burning)/aggravation by physical activity.

^b^Photophobia/phonophobia/nausea

^c^visual/sensory

^d^Assessment initiated 4h post infusion

na – not applicable

**Supplemental Table 3. Adverse events and medication intake in women with migraine under atosiban and placebo**

| **ID** | **Provocation** | **Time** | **Adverse events** | **Aura symptoms** | **Medication** |
| --- | --- | --- | --- | --- | --- |
| **113** | Atosiban (1) | 4-7h | Dry mouth; fatigue |  |  |
|  | Placebo (2) | 5h |  |  | Sumatriptan 100mg |
|  |  | 6h | Motor weakness with arm predominance |  |  |
|  |  | 11h |  |  | Sumatriptan 50mg |
| **114** | Placebo (I) | 70min | Palpitations |  |  |
|  |  | 11h |  | Visual aura |  |
|  |  | 12h | Tinnitus |  |  |
| **115** | Atosiban (1) | 10min | Feeling of warmth |  |  |
|  |  | 40-80min |  |  |  |
|  |  | 2h |  |  |  |
|  |  | 5h |  |  | Ibuprofene 800mg |
|  |  | 12h |  |  | Ibuprofene 800mg |
| **117** | Placebo (2) | 11h |  |  | Ibuprofene 600mg |
| **121** | Atosiban (1) | 30min | Hyperosmia |  |  |
|  |  | 4h | Feeling of warmth |  |  |
|  |  | 8-9h |  |  |  |
|  |  | 4-6h |  | Visual aura |  |
|  |  | 4h  8h |  | Sensory aura |  |
|  |  |  |  |  |  |
|  |  | 4h | Altered body perception |  |  |
|  |  | 8h |  |  |  |
|  | Placebo (2) | 4-7h | Palpitations; feeling of warmth |  |  |
|  |  | 4-7h |  | Visual aura |  |
|  |  | 4-7h |  | Sensory aura |  |
|  |  | 4h | Syncope; hearing and vision loss |  |  |
|  |  | 4-12h | Pain in the limbs |  |  |
| **123** | Placebo (I) | 2h |  |  | Ibuprofene 600mg |
|  |  | 5h | Palpitations |  |  |
|  |  | 7h |  |  |  |
|  |  | 6h |  |  | Sumatriptan 100mg |
|  |  | 10-11h | Fatigue; weakness; “hangover” |  |  |
| **125** | Atosiban (2) | 5-6h | Feeling of warmth |  |  |
|  | Placebo (I) | 4-6h | Feeling of warmth |  |  |
| **126** | Atosiban (1) | 7h |  |  | Rizatriptan 10mg |
| **127** | Atosiban (1) | 5-8h | Mild dizziness |  |  |
| **129** | Atosiban (1) | 170min | Mild dizziness |  |  |
| **130** | Atosiban (1) | 80-100min | Mild tension |  |  |
|  |  | 110min |  |  |  |
|  |  | 4h | Tension in neck and jaw |  |  |
|  |  | 11-12h |  |  |  |
|  |  | 11-12h |  | Sensory aura |  |
|  | Placebo (2) | 4-5h | Slight feeling of tension in right forehead/temple |  |  |
| **132** | Atosiban (1) | 4h |  |  | Ibuprofene 600mg |
| **133** | Atosiban (1) | 60min | Mild ear pressure (“ringing in the ears”) |  |  |
|  |  | 70min | Increasing ear pressure |  |  |
|  |  | 100 min | Mild dizziness; right periorbital head pressure |  |  |
|  |  | 5h | Redness; Feeling of warmth |  |  |
|  |  | 7h |  |  | Paracetamol 500mg |
|  | Placebo (2) | 5-9h | Feeling cold |  |  |
| **134** | Atosiban (2) | 0min | Sudden, brief nausea after bolus administration |  |  |
|  |  | 170min | Subjective right-sided weakness; mild ptosis |  |  |
|  |  | 9h |  |  | ASS 500mg |
|  |  | 9-10h | Feeling of warmth |  |  |

**Supplemental Table 4. Adverse events and medication intake in men with migraine under atosiban and placebo**

| **ID** | **Provocation** | **Time** | **Adverse events** | **Aura symptoms** | **Medication** |
| --- | --- | --- | --- | --- | --- |
| **201** | Placebo (2) | 8-9h | Dizziness; lightheadedness |  |  |
|  |  | 9 |  |  | ASS 1000mg |
|  |  | 10-12h | Mild dizziness |  |  |
| **202** | Atosiban (1) | 160min |  | Visual aura |  |
| **207** | Atosiban (2) | 70min |  | Visual aura |  |
| **209** | Placebo (2) | 50-60min | Malaise |  |  |
|  |  | 8h |  |  | Ibuprofen 400mg |
| **210** | Atosiban (1) | 80-130min | Mild dizziness |  |  |
| **211** | Atosiban (2) | 0h | Sudden, brief nausea after bolus administration |  |  |
|  | Placebo (I) | 40-60min | Mild neck tension |  |  |
|  |  | 110-180min | Occipital pressure/tension sensation |  |  |
|  |  | 4h |  |  | Ibuprofen 400mg |
|  |  | 6h |  |  | Ibuprofen 400mg |
|  |  | 7h |  |  | Ibuprofen 400mg |
| **213** | Placebo (I) | 4-6h | Malaise |  |  |
| **214** | Atosiban (2) | 10min | Mild dizziness; malaise |  |  |
|  |  | 20min | Feeling of warmth |  |  |
|  |  | 30-180min | Redness |  |  |
|  |  | 170-180min | Feeling of warmth |  |  |
| **216** | Atosiban (1) | 4h |  | Sensory aura |  |
| **217** | Atosiban (1) | 0h | Sudden, brief nausea after bolus administration |  |  |
| **222** | Atosiban (2) | 7h |  |  | Ibuprofen 400mg |
| **224** | Atosiban (1) | 0h | Severe dizziness |  |  |
|  |  | 40-50min | Mild dizziness |  |  |
|  |  | 120-140min | Severe dizziness |  |  |
|  |  | 8h | Mild dizziness |  |  |
|  | Placebo (2) | 7h |  |  | Ibuprofen 400mg |

**Supplemental Table 5. Adverse events and medication intake in healthy female controls under atosiban and placebo**

| **ID** | **Provocation** | **Time** | **Adverse events** | **Aura symptoms** | **Medication** |
| --- | --- | --- | --- | --- | --- |
| **309** | Atosiban (2) | 0min | Redness; feeling of warmth |  |  |
| **313** | Atosiban (1) | 4h | Feeling of warmth |  |  |
|  |  | 9-12h |  |  |  |
|  | Placebo (2) | 4h | Feeling of warmth |  |  |
|  |  | 9h |  |  | Paracetamol 500mg |
| **315** | Placebo (I) | 8h |  | Visual aura |  |
| **320** | Placebo (I) | 7-10h | Feeling of warmth |  |  |
| **324** | Atosiban (2) | 9-12h | Mild rhinorrhea; sensation of having a cold |  |  |
| **327** | Atosiban (1) | 0h | Sudden, brief nausea after bolus administration; altered taste perception (bitter) |  |  |
| **330** | Placebo (2) | 8h | Diarrhea |  |  |
